# Development of polarization-sensitive optical coherence tomography imaging platform and metrics to quantify electrostimulation-induced peripheral nerve injury *in vivo* in a small animal model

**DOI:** 10.1117/1.NPh.10.2.025004

**Published:** 2023-04-17

**Authors:** Guillermo L. Monroy, Mohsen Erfanzadeh, Michael Tao, Damon T. DePaoli, Ilyas Saytashev, Stephanie A. Nam, Harmain Rafi, Kasey C. Kwong, Katherine Shea, Benjamin J. Vakoc, Srikanth Vasudevan, Daniel X. Hammer

**Affiliations:** a U. S. Food and Drug Administration, Center for Devices and Radiological Health, Office of Science and Engineering Laboratories, Division of Biomedical Physics, Silver Spring, Maryland, United States; b Massachusetts General Hospital, Harvard Medical School, Wellman Center for Photomedicine, Boston, Massachusetts, United States; c Harvard Medical School, Boston, Massachusetts, United States; d U. S. Food and Drug Administration, Center for Drug Evaluation and Research, Office of Clinical Pharmacology, Office of Translational Science, Division of Applied Regulatory Science, Silver Spring, Maryland, United States; e Massachusetts Institute of Technology, Division of Health Science and Technology, Cambridge, Massachusetts, United States

**Keywords:** neuromodulation, nerve stimulation, peripheral nerve injury, polarization-sensitive optical coherence tomography, optical metrics, rat, sciatic nerve

## Abstract

**Significance:**

Neuromodulation devices are rapidly evolving for the treatment of neurological diseases and conditions. Injury from implantation or long-term use without obvious functional losses is often only detectable through terminal histology. New technologies are needed that assess the peripheral nervous system (PNS) under normal and diseased or injured conditions.

**Aim:**

We aim to demonstrate an imaging and stimulation platform that can elucidate the biological mechanisms and impacts of neurostimulation in the PNS and apply it to the sciatic nerve to extract imaging metrics indicating electrical overstimulation.

**Approach:**

A sciatic nerve injury model in a 15-rat cohort was observed using a newly developed imaging and stimulation platform that can detect electrical overstimulation effects with polarization-sensitive optical coherence tomography. The sciatic nerve was electrically stimulated using a custom-developed nerve holder with embedded electrodes for 1 h, followed by a 1-h recovery period, delivered at above-threshold Shannon model k-values in experimental groups: sham control (SC, n=5, 0.0  mA/0  Hz), stimulation level 1 (SL1, n=5, 3.4  mA/50  Hz, and k=2.57), and stimulation level 2 (SL2, n=5, 6.8  mA/100  Hz, and k=3.17).

**Results:**

The stimulation and imaging system successfully captured study data across the cohort. When compared to a SC after a 1-week recovery, the fascicle closest to the stimulation lead showed an average change of +4%/−309% (SL1/SL2) in phase retardation and −79%/−148% in optical attenuation relative to SC. Analysis of immunohistochemistry (IHC) shows a +1%/−36% difference in myelin pixel counts and −13%/+29% difference in axon pixel counts, and an overall increase in cell nuclei pixel count of +20%/+35%. These metrics were consistent with IHC and hematoxylin/eosin tissue section analysis.

**Conclusions:**

The poststimulation changes observed in our study are manifestations of nerve injury and repair, specifically degeneration and angiogenesis. Optical imaging metrics quantify these processes and may help evaluate the safety and efficacy of neuromodulation devices.

## 1 Introduction

Therapeutic electrical neuromodulation devices are under active development for the treatment of numerous diseases and conditions,^1^^,^^2^ including epilepsy, depression, digestion/bowel function, hunger regulation,^3^^–^^6^ rheumatoid arthritis, and Crohn’s disease.^7^^,^^8^ However, electrode implantation, unsafe stimulus parameters, prolonged application of electrical stimulation, or constriction of local nerve blood flow, can all potentially damage the nerve, cause loss of function, or result in reduced treatment effectiveness.^9^^,^^10^ Disease states further complicate assessment of neural activity and behavior, as many pathways are not fully understood, even under healthy conditions. New tools and biomarkers are needed to characterize the function of the peripheral nervous system (PNS) as well as validate the safety and performance of neuromodulation devices.

The sciatic nerve is often used as a model for PNS injury,^11^ with many studies exploring different parameters for safe delivery of electrical stimulation,^12^^,^^13^ the effect of nerve compression,^14^^,^^15^ and transection injuries.^16^^,^^17^ In contrast to mechanical trauma or thermal injuries, electrically stimulated nerves may appear visibly and structurally intact but exhibit measurable functional deficits. For example, post-injury weakness and fatigue have been reported in clinical cases of electrical shock and lightning strike.^18^ Generally, damage in the PNS is thought to be caused in distinct stages,^19^^,^^20^ where acute onset is related to direct nerve damage and delayed onset is related to inflammation and edema. Histological evaluation of electrically damaged nervous tissue often shows damage directly to axons and myelin.^10^ However, many of these studies required invasive sampling of one or multiple animals per timepoint, which precludes real-time or single-subject longitudinal monitoring.^21^ There is a need for real-time *in vivo* approaches where nerve injury can be tracked and characterized over time.

Optical coherence tomography (OCT) has gained substantial interest in many clinical disciplines as a noninvasive diagnostic tool, especially in ophthalmology and cardiology.^22^ OCT provides quantitative, high-speed, and high-resolution (<10  μm) cross-sectional and volumetric scans of tissue. OCT is unique among optical imaging modalities, as the axial resolution is dependent on the source bandwidth rather than the numerical aperture of the imaging objective. This allows micron-level axial resolution and cross-sectional optical biopsies of tissues. OCT has many associated submodalities that provide a wealth of information relevant to neuromodulation of the PNS. These include reflectance (structural OCT),^23^^–^^26^ blood flow [OCT angiography (OCT-A)],^27^^,^^28^ and more recently, birefringence [polarization-sensitive OCT/(PSOCT)].^29^^–^^31^

By combining multiple contrast mechanisms, OCT imaging provides a means of assessing healthy, diseased, and damaged whole nerve tissues and distal organ targets without the use of exogenous dyes or labels. Noninvasive OCT imaging enables the observation of longitudinal effects of a specific therapy within a single animal. Relatively few studies have applied OCT to observe PNS injury and repair^32^ or the effect of electrical stimulation injuries in the PNS.^33^ A previous study from our group demonstrated blood vessel dilation and flow rate increase with electrical stimulation using OCT.^34^ Recent work observed changes in birefringence and perfusion with nerve crush injury using PSOCT, where damage was longitudinally tracked in a single animal.^35^

In this study, a custom-designed imaging and stimulation platform was developed to assess the effect of electrical overstimulation in a rat sciatic nerve injury model. OCT-based metrics representative of longitudinal changes to the structure (OCT), birefringence (PSOCT), and perfusion (OCT-A) of the nerve were quantified and compared with histology. These metrics were developed and compared under healthy and electrically stimulated conditions relevant for neuromodulation over the course of multiple days. Optical metrics were then analyzed and compared to histological findings.

## 2 Methods

### 2.1 Imaging and Stimulation System

**Fig. 1:**
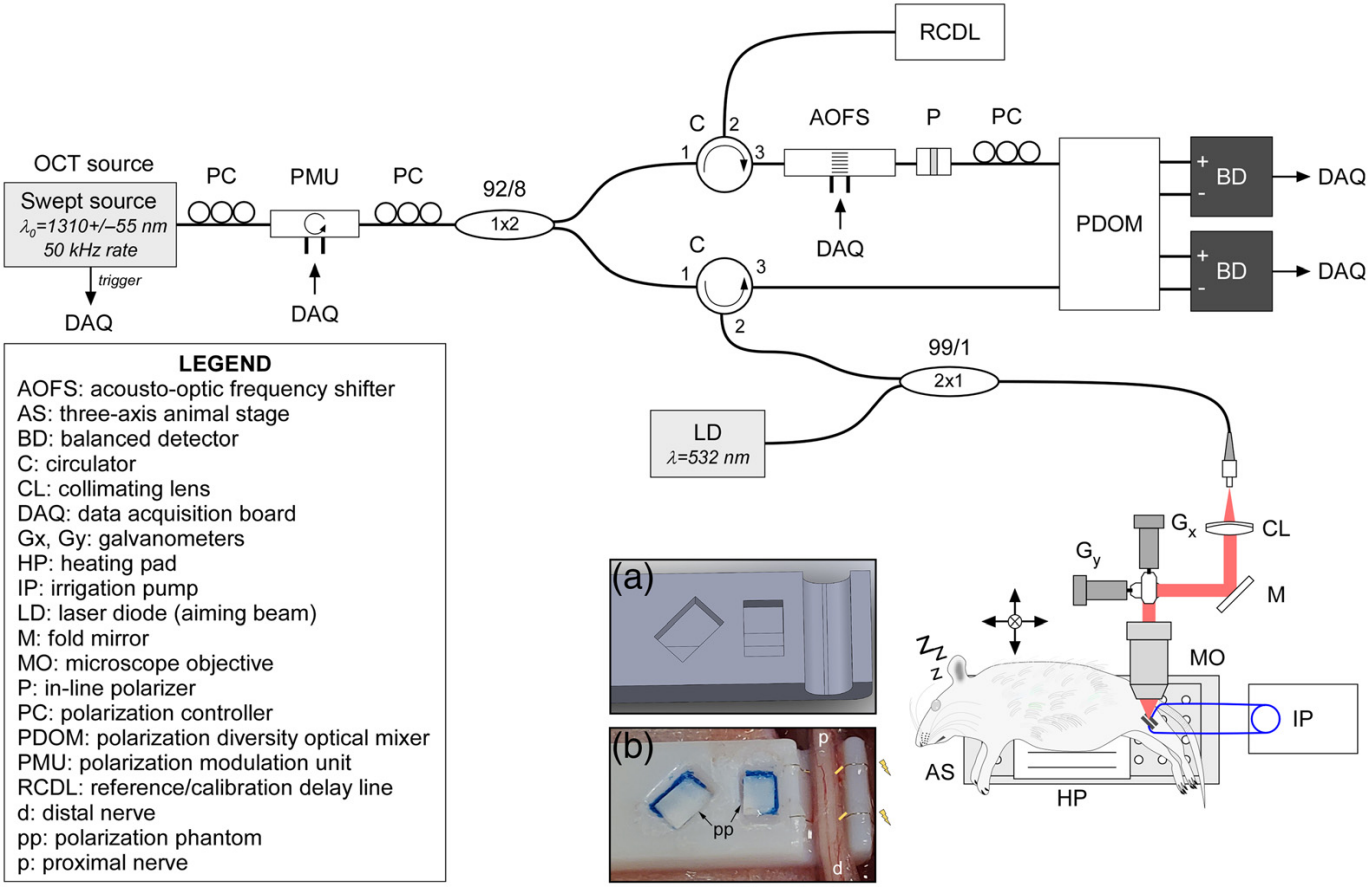
Optical coherence tomography (OCT) imaging: full system layout. This custom-built swept-source polarization-sensitive OCT system was designed to capture volumetric scans of the sciatic nerve during electrical stimulation. This system noninvasively extracts tissue features (structure, birefringence, and perfusion) without stains or dyes. (a) Nerve stabilizer 3D-model and (b) stabilizer with sciatic nerve *in situ.* Deinsulated portions of the electrodes are noted with yellow bars, nearest to the rightmost fascicle.

A custom-developed polarization-sensitive optical coherence tomography (PSOCT) imaging and stimulation platform, shown in detail in Fig. 1, was developed and optimized to observe and characterize PNS tissue during and after recovery from electrical stimulation. The system used a near-infrared (1310 nm) swept-source laser focused into tissue to generate depth-resolved optical scattering profiles. A 110-nm bandwidth swept-source laser (HSL-200-50LC, Santec Corp., Japan) provided an axial and lateral resolution of ∼7 and 20  μm (in air). Three-dimensional imaging was achieved by scanning the imaging light across tissue using a pair of galvanometer scanners. A green tracking laser was added to help operators visualize the scan location incident on tissue. To measure birefringence independent of optical axis, this Jones matrix-based system modulates the input polarization state of light on alternating A-lines^36^ using a custom-fabricated semiconductor-based polarization modulator (Boston Applied Technologies, United States). OCT-A scans are simultaneously collected with PSOCT data by taking five sequentially repeated scans at each physical location. All OCT imaging was performed without the use of exogenous stains or dyes, and each dataset was collected in ∼2  min.

A custom-designed 3D-printed nerve stabilizer was modified from the previous work^34^ to include PSOCT calibration phantoms^37^^–^^39^ and embedded stimulation electrodes [Figs. 1(a) and 1(b)]. The stabilizer supports the nerve during imaging to reduce motion artifacts from breathing and electrical stimulation. The pair of phantoms, further described in Fig. S2 in the Supplementary Material, serves as external markers to calibrate the optic axis of the imaging system at 0° and 45° with respect to each other. These fixed external references allow measurements to be directly compared to one another, even when collected on different days or even different imaging systems. For stimulation, two Teflon-coated platinum/iridium hemi-cuff stimulation electrodes were installed (Microprobes for Life Science, United States). Both electrodes had a diameter of ∼0.05  mm, with deinsulated regions of 0.2 mm (positive) and 0.4 mm (negative) within the nerve stabilizer holder. A MATLAB-controllable precision AC/DC current source (6221, Keithley Instruments, United States) delivers customizable stimulation waveforms. To reduce the appearance of bands of Fontana, a rodent leg extender (RLE) was created. The RLE, discussed in further detail in Fig. S3 in the Supplementary Material, gently and safely applies force around the ankle using a rubber band to pull the leg and sciatic nerve into its naturally extended position. A nearby syringe pump administered sterile saline (0.05  mL/min) over the course of experiments to keep tissue hydrated. Finally, a sterile, thin piece of plastic film, approximately the dimensions of the nerve channel, was placed over the nerve to reduce back reflections and prevent dehydration effects over the imaging period. Custom-develop GPU-based processing and polarimetry-reconstruction code^40^ was developed to process raw data, account for system-specific noise, and accurately reconstruct tissue morphology in PSOCT images. A more complete description of the imaging and stimulation platform design, specifications, and testing is available in the Supplementary Material.

**Fig. 2:**
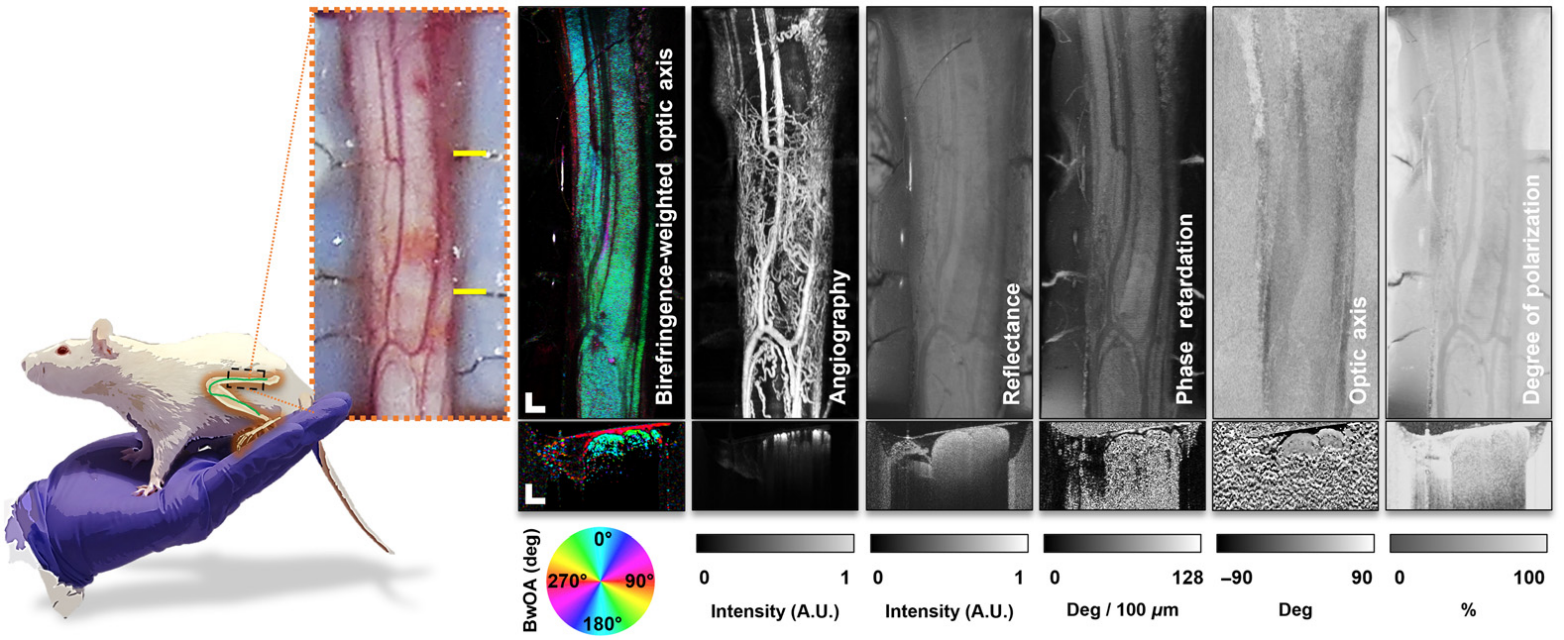
Platform output: processed PSOCT data, animal #2: SC, D7. Representative data from a rat sciatic nerve as observed in this study of the six data channels captured. *En face* representations and cross-sectional scans for each channel are shown. BwOA, birefringence-weighted optic axis. Scale bar: 500  μm.

From the OCT signal, six distinct imaging outputs (channels) were calculated from each volumetric acquisition. Together, these six channels quantify a range of tissue optical properties. Imaging metrics that characterize the optical and birefringent properties of tissue and vessel perfusion were compared across treatment groups during stimulation and after recovery. The six channels are illustrated in Fig. 2. Birefringence-weighted optic axis (BwOA) is derived from a combination of phase retardation and optic axis^39^ and provides a color-coded visualization of the vectorial birefringence, effectively the orientation of the microstructure of tissue. Angiography (OCT-A) is a tissue vascular map and is extracted from temporal signal fluctuations due to blood flow. Reflectance data, the typical structural representation of OCT data, derives contrast from the scattering magnitude of various tissues. Phase retardation and optic axis are both related to the birefringent or optical polarization properties of tissue. Finally, the degree of polarization uniformity (DOPU) measures the consistency of tissue properties as the imaging beam propagates through tissue. In aggregate, these channels are sensitive to both the structural, physiological, and birefringent optical properties of tissue.

### 2.2 Experimental Design and Protocol

Animal work performed in this study was approved and conducted with oversight by the U.S. FDA (White Oak campus) Institutional Animal Care and Use Committee. 15 female Lewis rats (279±12  g, Charles River Laboratories, Inc., United States) were acquired for this study. Upon arrival, all subjects were allowed a 5-day acclimation period with 12 h day/night cycles before experimental use.

**Fig. 3:**
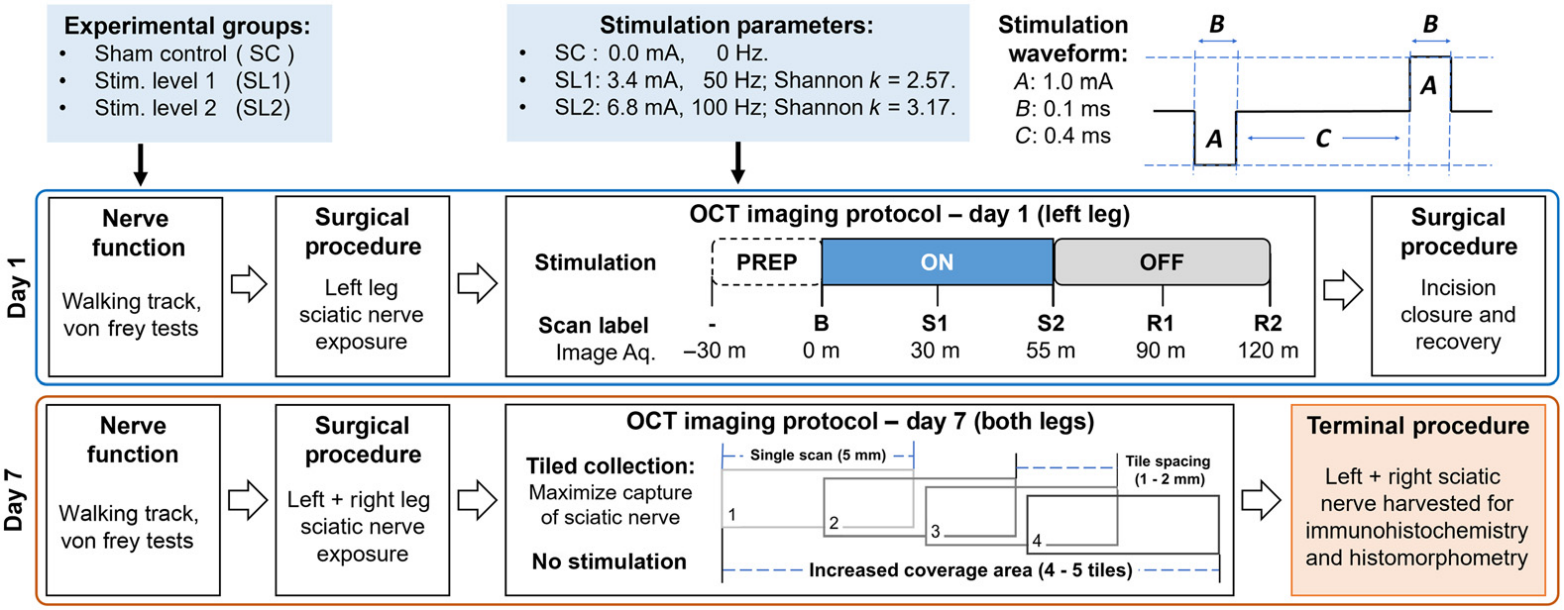
Surgical and imaging experimental protocol. Day 1 began with a baseline assessment of nerve function, followed by sciatic nerve exposure surgery. Afterward, the animal was placed into the imaging setup for a 30-min acclimation period (PREP). A baseline (B) OCT scan of the nerve was captured before stimulation. Stimulation was active for 1 h (ON) followed by a 1-h recovery (OFF). Simultaneously, OCT scans of the stimulation region were collected every 30 min (during stimulation: S1, S2; and recovery: R1, R2). On day 7, nerve assessment tests were repeated, and the left sciatic nerve re-exposed for imaging. OCT scans were collected using a tiled collection strategy to ensure capture of the stimulation region from day 1. Nerve exposure and tiled imaging was repeated on the contralateral (right) leg to serve as an internal control. Finally, the sciatic nerves from both legs were harvested for histological analysis.

Figure 3 shows an overview of the experimental protocol used for this study. Surgery and imaging were performed by the same team to limit experimental variance. The animals (n=15) were equally and randomly assigned into three groups: sham control (SC, n=5, 0.0  mA/0  Hz), stimulation level 1 (SL1, n=5, 3.4  mA/50  Hz, Shannon k=2.57), and stimulation level 2 (SL2, n=5, 6.8  mA/100  Hz, Shannon k=3.17). For stimulation, two Teflon-coated platinum/iridium hemi-cuff stimulation electrodes were installed into the nerve channel (Microprobes for Life Science, United States). Both electrodes had a diameter of ∼0.05  mm, with deinsulated regions of 0.2 mm (positive) and 0.4 mm (negative) within the nerve stabilizer holder. The stimulation waveform used here is a cathodic-first charge-balanced square wave (100  μs pulse width, 400  μs interphase delay), with damage thresholds identified using the Shannon model.^41^ Shannon k values above ∼1.85 have been shown to cause damage,^12^ with higher values producing more severe damage. For the SC group, the output of the stimulation system was connected via electrodes to the animal, but the output was set to 0.0  mA/0  Hz.

On day 1 (D1), a functional baseline nerve assessment for each animal was collected immediately prior to experimentation using both walking track^42^ and Von Frey analyses.^43^ To collect a measure of gait, specifically through sciatic function index (SFI) and tibial function index (TFI), each animal was placed onto a clear plexiglass platform (FreeWalk, CleverSys, United States) and restricted within an 8  cm×40  cm rectangular area.^44^ At least five continuous steps for each foot were recorded using a digital video camera. Afterward, animals were transferred to a plexiglass enclosure with a perforated metal floor (25.4  cm×25.4  cm) for a Von Frey assay. Animals were acclimated within the setup for 5 min, and then a rigid filament esthesiometer was used to measure 5 trials on both hind limbs by applying centered point-stimulation on the plantar surface of the paw. Forces resulting in a clear paw withdrawal were recorded for each foot.

Animals were then anesthetized and prepared for surgery. The complete surgical method is described in the Supplementary Material. During surgery, animals were thermally stabilized, fully anesthetized, and hydrated during and after the procedure. After surgery, animals were transferred to the imaging and stimulation platform and loaded into the RLE and nerve stabilizer (see Fig. S3 in the Supplementary Material for more details) for a 30-min acclimation period. Animals were stimulated for 1 h, following the parameters specified for each group, then allowed to recover for 1 h. Simultaneously, PSOCT volumes of the stimulation region were collected, starting with a baseline scan collected just prior to stimulation, and then every 30 min thereafter. The second scan was taken at 55 min to avoid scan acquisition during the stimulation system shutoff at 60 min, which can otherwise lead to movement artifacts. A digital photograph of the nerve within the holder was taken immediately prior to the baseline scan and after recovery to assist with nerve identification. Animals were returned to the surgical microscope field to close the incision and recover from anesthesia. Postsurgical antibiotics and analgesics were given to each animal.

On day 7 (D7) after the recovery period, functional assessments were repeated, and the left sciatic nerve was surgically re-exposed. Overlapping PSOCT volumes of the nerve were collected to capture the original stimulation region and surrounding nerve tissue. Capturing 4 to 5 tiled volumes ensured acquisition of the stimulation region and aided in co-registration between D1/D7. Digital photographs of the nerve at each imaging location were also taken. After imaging the left leg, sciatic nerve exposure surgery followed by the D7 imaging protocol was repeated on the contralateral (right) leg. Finally, the sciatic nerves from both legs were harvested for histological processing and analysis.

### 2.3 Histological Processing

Each nerve was separated into three segments, relative to where stimulation was applied and placed into separate vials with a fixative agent for histology. Distal and proximal segments (∼2 to 3 mm length portions) from the stimulated nerve region (e.g., closer to hip or limb extremity) were outside of the OCT scan area. The median segment was ∼7 to 10 mm in length and known to contain the stimulated nerve region with confirmed representation in PSOCT image data. Using surgical photographs and OCT *en face* reconstructions, the stimulation region was identified in the median segment and further sectioned prior to paraffin embedding. Median segments, after this sectioning, were ∼4 to 5 mm. Median segments were fixed in 4% paraformaldehyde, rinsed in phosphate-buffered saline (PBS), embedded in paraffin blocks (ASPS300S, Leica Biosystems), and manually sectioned into 5  μm sections. Two slides, each containing three sections, were taken from the median segment for immunohistochemistry (IHC). One set of cross sections was stained with hematoxylin and eosin (H+E) for coarse analysis.

For IHC, sections were put into a 60°C oven for 30 min, rehydrated in 100% xylene and several ethanol solutions (100%, 90%, and 70%), and incubated in citrate buffer antigen retrieval solution. Afterward, slides were incubated in a 4% blocking solution of goat serum (Thermo Fisher Scientific) and washing solution [0.5% Triton X-100 (Sigma) in PBS] for 1 h at room temperature and then incubated overnight with primary antibodies at 4°C. Myelin protein zero (P0) and β-tubulin-III were used as primary antibody targets to observe the myelination and axon changes in the nerve. The primary antibodies used were rabbit anti-P0 (1:150; Millipore) and mouse anti-β-tubulin-III (1:150; Sigma) immunoglobulin G1 antibodies (IgG1). Slides were then rinsed and incubated in a secondary antibody for 1 h at room temperature. These steps were carried out in the dark to prevent photobleaching of the secondary antibodies. Fluorescent secondary antibodies used were goat anti-mouse-IgG1 (1:100; Jackson ImmunoResearch) and goat anti-rabbit-IgG (1:100; Jackson ImmunoResearch). These secondary antibodies were tagged with AlexaFluor 488 and AlexaFluor 594, respectively. After a final incubation, excess washing solution was removed, and slides were mounted with gold antifade mount containing DAPI (Thermo Fisher). DAPI was used as a nuclear counterstain. Slides were then imaged on an FV 1000 confocal microscope (Olympus) at 10× magnification.

### 2.4 Data Extraction and Analysis Methods

#### 2.4.1 Walking track and Von Frey assays

Videos of animal gait on the walking track were manually analyzed frame-by-frame in ImageJ software to isolate individual steps and measure foot and toe spread parameters. These parameter measurements included: left footprint length, left toe spread, left intermediary toe spread, right footprint length, right toe spread, and right intermediary toe spread. These parameters were used to calculate SFI and TFI, as described previously.^34^ Functional index values and Von Frey measurements were analyzed using two-way ANOVA, Tukey’s multiple comparison test, and Bonferroni’s multiple comparison test in MATLAB (MathWorks Inc.). Correlation between the index values and the SL or the gait and timepoint (pre- and postprocedure) were analyzed. Correlation between value and measurement site [ipsilateral (treatment/stimulated) or contralateral leg], were accounted for along with possible interactions with stimulation group and/or timepoint.

#### 2.4.2 PSOCT data processing, co-registration, and analysis

**Fig. 4:**
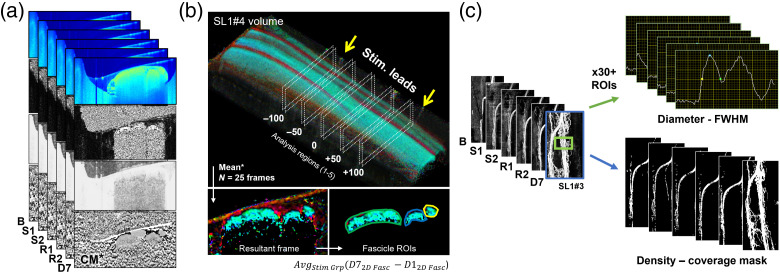
Data extraction methods. (a) Data channels were organized and co-registered across each imaging timepoint for analysis (from top to bottom: intensity, phase retardation, DOPU, and optic axis). (b) A 3D representation of nerve volume is plotted using BwOA channel. ROI selection: five analysis regions within the stimulation region were selected to extract representative frames. At each location, 25 frames were averaged to generate a resultant frame. An arithmetic or coherent mean (CM) was used to ensure data integrity during processing. For each of the five resultant averaged frames, ROIs were manually segmented using histology and PSOCT image morphology to select only the fascicle (teal) and not the epi- or perineurium (red). From each ROI, a mean value for each fascicle can be extracted and group statistics computed. (c) To analyze angiography data, the full *en face* dataset from each timepoint was processed to extract both vessel diameter and density metrics. Changes in vessel diameter (top) were calculated by manually selecting at least 30 segments per timepoint, extracting the mean FWHM. To compute vessel fraction (bottom), a coverage mask was computed. A ratio (%) of vessel to total (vessel + background) pixels is calculated for each frame.

The data organization, processing, and ROI selection are visualized in Fig. 4. With 6 channels, 15 animals, and 6 timepoints (n=6×15×6), the data library was first organized then co-registered across imaging timepoints. The optimal D7 tile was carefully chosen that best overlaps with scans from D1 using structural cues from the angiography map and overall fascicle structure, ensuring the stimulation region from D1 is fully visible in the D7 tile. Four of the six channels were chosen for in-depth analysis: attenuation maps (calculated from reflectance data^45^), phase retardation, DOPU, and optic axis [top to bottom, Fig. 4(a)]. These contrast mechanisms have been successfully used in other studies to distinguish between healthy and diseased tissues.^46^^–^^49^ For nervous tissue, electrostimulation-induced damage to myelination is of primary interest.

To capture ROIs representative of the data and to limit selection bias, five analysis regions in four data channels (n=5×4×15×6) were selected for each dataset in Fig. 4(b) (top, dotted boxes) that span the stimulation region within the electrodes of the D1 volume and overlap through all other timepoints. In each of the 5 regions, 25 cross-sectional frames were averaged (∼L×W×H:100  μm×2.5  mm×2  mm) into 1 resultant frame [Fig. 4(b), lower left). Depending on the data type/channel, a coherent mean (optic axis^39^^,^^50^) or standard arithmetic mean was used to maintain data integrity during processing. Within these five resultant frames, fascicle ROIs were then manually segmented based on structural cues cross-referenced from both PSOCT image morphology and histological sections [Fig. 4(b), lower right). Although a more complete extent of the nerve profile can be visualized in the reflectance channel, multiple scattering causes depolarization and degradation in polarization-sensitive information in deeper regions. ROI depth was thus conservatively chosen using PS data to avoid including invalid data or regions beyond the imaging depth of the system, which would detrimentally add noise during analysis. A unique ROI selection for each fascicle and for each timepoint was saved to ensure that any temporal changes in tissue have been captured. Fascicles were labeled based on proximity to the stimulation leads: left, center, and right [L/C/R, shown as green/blue/yellow in Fig. 4(b), lower right], where the rightmost region is closest to the stimulation leads (n=3×5×4×15×6). Finally, average values were computed using the entire fascicle 2D ROI. The statistics for D7 to D1 values were calculated for each animal and averaged within each experimental group for comparison. This calculation aims to remove animal–animal variability using D1 values as a baseline correction for changes at D7. Generally: $\Delta \text{metric}={\mathrm{avg}}_{\text{stim}\;\mathrm{grp}}(D7_{2D\;\text{fasc}}-D1_{2D\;\text{fasc}}$).

To analyze angiography maps [Fig. 4(c)], volumetric scans were converted into *en face* vessel map average intensity projections (∼400 to 600  μm in-depth to include signal from the entire nerve). Customized MATLAB scripts aligned the *en face* images using a cross-correlation and peak finding method for optimal co-registration across all timepoints, using major vessels as landmarks. Across D1 timepoints (B, S1, S2, R1, and R2), co-registration was straightforward, as movement was minimized using the nerve stabilizer and RLE. The co-registration of D7 data was challenging but feasible due to some slight rotation and compression/stretching of tissue between timepoints. Regions outside of the co-registered D1 to D7 region were cropped from the stack. Finally, horizontal lines caused by any motion artifacts were reduced using a local vertical profile smoothing method.^51^

Vessel diameter values from the dataset were manually extracted using a custom-developed LabVIEW program (National Instruments Inc.).^52^ The software operates on *en face* OCT-A images and using vessel ROIs set by the operator, extracts the full-width at half-maximum (FWHM) vessel diameter. Thirty vessel measurements were made for each timepoint. Vessel density coverage maps were extracted and quantified using a separate custom-developed LabVIEW program.^52^ This software also operates on *en face* OCT-A images and applies several filtering steps (scaling, thresholding, and morphological noise reduction operators) within a selected en face vessel density ROI to separately mask vessel and background pixels. The filtering settings and ROI were empirically determined to segment all vessels at all timepoints while also reducing the influence of spurious motion artifacts and noise. Within the density ROI, the ratio of the number of pixels within the vessel mask is compared to the total number of pixels in the density ROI (vessel + background) to provide a measure of vessel coverage (i.e., vessel density). With values extracted from every timepoint for these metrics, experimental changes were calculated by taking the D7 value referenced to D1 as a baseline. The results were averaged within each stimulation group for comparison and plotting.

#### 2.4.3 Nerve segmentation methods

OCT reflectance volumes were averaged laterally every ∼170  μm (∼40 cross-sectional frames along the nerve) and manually analyzed in Neurolucida360 (MBF Bioscience, United States). The tissue volume was measured by segmenting cross-sections and extruding the segments in Neurolucida360 to create a volume. *En face* images were used to carefully co-register physiological features when segmenting, nerve branches that extend beyond the nerve holder, and thicker areas of the tissue where the signal depth penetration becomes an issue. Nerve volumes were analyzed in MATLAB with two-way ANOVA, Tukey’s, and Bonferroni’s statistical tests.

#### 2.4.4 Histological analysis

Histological slides were co-registered with OCT data and analyzed for relevant markers of damage compared to SCs. Hematoxylin and eosin (H+E) slides were examined for changes to tissue organization and regularity, blood vessel presence and density, myelin sheath continuity, peri/epineurium, and other markers that may indicate nerve damage. To quantify IHC fluorescence images, fascicle ROIs were manually segmented and RGB channels, corresponding to myelin (red), axons (green), and cell nuclei (blue), were analyzed to extract a relative pixel count and compared to SCs. Proper histological processing techniques resulted in fluorescent images that were free from noise (RGB channel signal outside of the tissue) that could adversely affect this measurement. Instead of relying on brightness values in the image to quantify changes, which could vary from slide to slide, each channel was masked to identify only the presence or absence of fluorescence in each pixel. These counts were used to simplify analysis and reduce any influence from nonuniformity in the staining and imaging process.

## 3 Results

### 3.1 Surgical Results

Animals were closely monitored for 5 days after surgery, with additional analgesia provided for 2 to 3 days. One animal died unexpectedly prior to recovery from surgery. All data from this animal were excluded from the analyses. All other animals recovered without complication.

### 3.2 Imaging Results

**Fig. 5:**
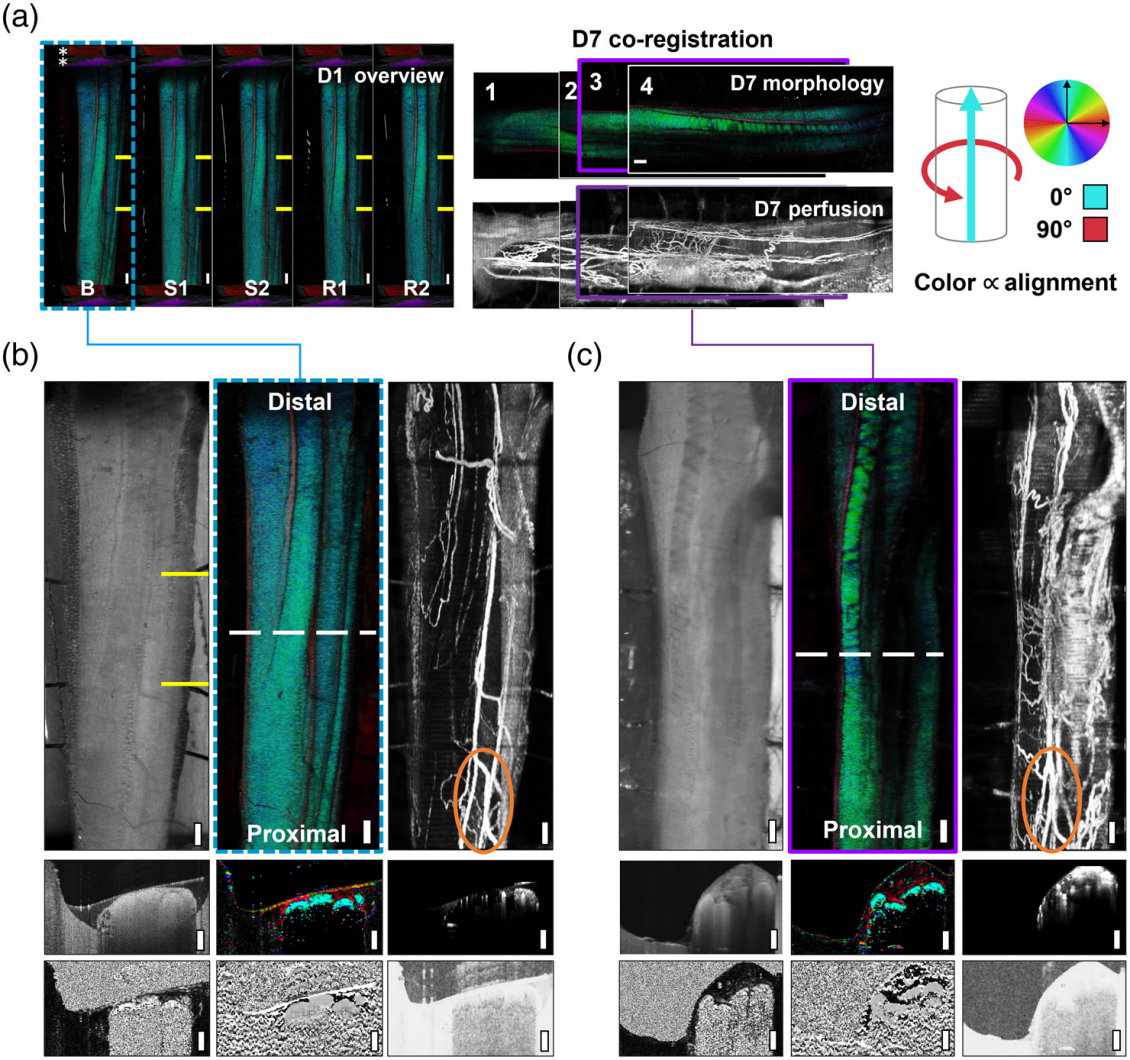
Representative imaging results, animal #4: SL1. (a) (left) OCT scans capture temporal dynamics of the left sciatic nerve during stimulation and recovery. Polarization phantoms (*, red/purple) shown in (a, left series), cropped in all other figures. (a, center): Overlapping scans are co-registered using cues from the structural organization of fascicles and perfusion maps. Tile #3 (purple, solid outline) corresponds best to day 1 scans (teal, dotted outline). BwOA colormap [(a), right] relates color to the physical orientation of tissue subunits. (b) Day 1 baseline, including *en face* projections of the structure (left), birefringent-weighted optic axis (BwOA, center), perfusion map (right), and cross-sectional scans (below) from all data channels. (c) Day 7 tile #3, co-registered data showing changes after a 7-day recovery. Note: yellow lines mark electrodes and stimulation region (day 1). Orange circles demonstrate one perfusion map visual cue for co-registration. White-dotted lines denote matching locations in cross-sectional data. Cross-sectional scans, from corresponding *en face* projections: (row 1) structural, BwOA, and angiography/perfusion and (row 2) phase retardation, optic axis, and degree of polarization uniformity. Distal and proximal labels indicate sciatic nerve orientation. Scale bars (white, solid): 500  μm.

Representative images from stimulation group level 1 (animal #4) are shown in Fig. 5. The complete dataset collected for this animal, encompassing BwOA D1 and co-registered D7 tiles, is shown in Fig. 5(a). It is possible to observe the calibration phantoms (4A/upper right *, cropped in all other panels), which are captured at the start and end of every scan. The stimulation lead location is marked in yellow on D1, and on D7 they were not active. The BwOA colormap links tissue orientation with color. The use of 0 deg and 45 deg polarization phantoms allowed a precise standardization of the presentation of the optic axis orientation across imaging timepoints and animal subjects.^37^ Figure 5(b) shows all data channels for the baseline (B) timepoint, whereas Fig. 5(c) shows the co-registered tile from D7 (purple boundary). As visually demonstrated in Fig. 3, overlapping tiles were collected of the nerve on D7 to capture the original stimulation region and surrounding tissue. D7 scans were then co-registered to one another and to D1 scans. Angiography perfusion maps helped to identify the stimulation region from D1, even though some rotation and compression of the nerve occurred between D1 and D7. ROI regions were then carefully drawn to compare the same fascicle and region across timepoints. In this manner, data across these timepoints can be compared and quantified, capturing both temporal dynamics during stimulation on D1, and electrostimulation-induced changes after a 1-week recovery.

This dataset can be examined qualitatively for visual changes across D1 and D7. Across D1 timepoints from baseline to recovery, no changes in the structure or birefringence of the tissue were observed due to SLs used at above-threshold values. No severe or immediate changes to tissue were expected, which is confirmed in the observed data in D1 and compared to more severe stimulus, such as nerve crush models as previously demonstrated.^35^ At D7, a visible increase in nerve diameter was observed, likely caused by postsurgical and poststimulation swelling in the epineurium. While swelling and increased vascularization were observed overall, the relative extent or lack of swelling and vascularization was a function of the inherent biological variability between the animals. At D7 and within the stimulation region from D1 [Fig. 5(c)], an increase in vessel density was observed, as well as a loss of “BwOA” signal, visible both in the *en face* and cross-sectional images. This signal loss was seen only in the optic axis and phase retardation channels. At equivalent points along the nerve in other channels, such as reflectance, there was no loss in signal, indicating this is a result of a change in birefringence (tissue) and not because of low SNR, absorption, or other artifacts. Representative 2D cross-sectional images and 1D depth profiles from all three experimental groups are comprehensively presented in Fig. S6 and S7 in the Supplementary Material.

**Fig. 6:**
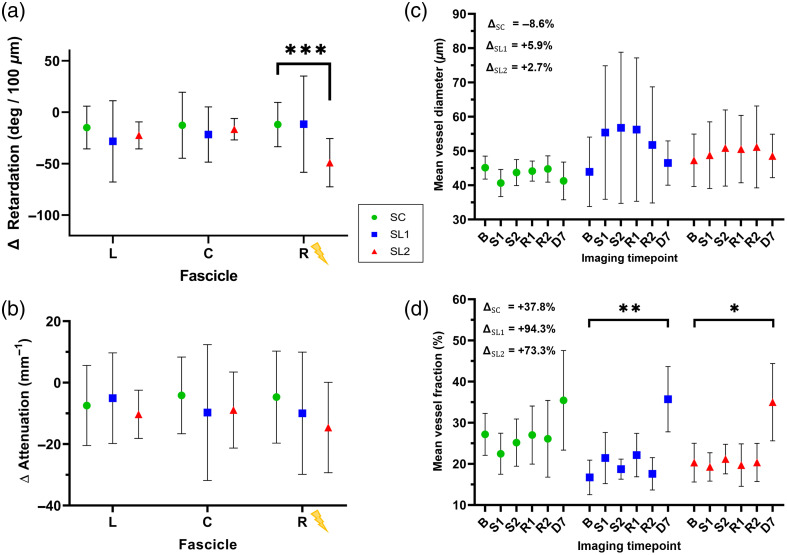
OCT metrics: changes after 1 week. (a) Phase retardation, D7 to D1. The rightmost fascicle nearest the stimulation electrode has the greatest effect, with the SL2 group with a significant reduction in phase retardation average value (SL2, ***p<0.001) after recovery. (b) Optical attenuation, D7 to D1. Results show a decrease in mean attenuation in the center and right fascicles in both SL1 and SL2. Left/center/right (L/C/R) denotes fascicle position, with R closest to the deinsulated portion of the electrode. (c) Blood vessel diameter (whole nerve), all timepoints across D1 and D7. Mean vessel diameter results demonstrate the stimulation group values increase in diameter under stimulation as expected and slowly decrease during the recovery period. Notably, a small increase over baseline values persists at 1 week for SL1 and SL2. (d) Blood vessel fraction (coverage, whole nerve), all timepoints across D1 and D7. Vessel fraction results show no appreciable change in the number of vessels over the course of day 1 as expected, though an increase in the total amount of vessels is seen at day 7 (SL1 **p<0.005 and SL2 *p<0.05). Error bars: ±SD. $\Delta \text{Metric}={\mathrm{avg}}_{\text{stim}\;\mathrm{grp}}(D7_{2D\;\text{fasc}}-D1_{2D\;\text{fasc}}$).

Figure 6 shows final results of OCT metrics separated by stimulation group (and fascicle for phase retardation and attenuation). For any data measuring a change, $\Delta \text{metric}={\mathrm{avg}}_{\text{stim}\;\mathrm{grp}}(D7_{2D\;\text{fasc}}-D1_{2D\;\text{fasc}})$. D7 to D1 differences were averaged for each fascicle and group and two-way ANOVA and Tukey’s tests applied to determine significance. For angiography values, mean values for each timepoint are shown to demonstrate the time-dependent nature of these effects, with repeated measures ANOVA and Tukey’s test applied to determine significance.

A significant decrease was observed in the phase retardation [Fig. 6(a)] in the SL2 stimulation group compared to SC in the rightmost fascicle. The SL1 group largely follows this trend, although one animal had larger phase retardation values than others, which diminishes the decrease. For optical attenuation [Fig. 6(b)], the right-most fascicle closest to the stimulation lead had the greatest change, although it did not reach statistical significance. Angiography data followed expected trends. During stimulation on D1, vessels dilated across the entire nerve, leading to a measured increase in diameter, but, not density, as seen previously.^34^ At D7, a small increase from baseline vessel diameter is retained in the stimulation groups, which was unexpected, as well as a large increase in the vessel density at D7 after recovery in all groups, with a larger increase in density in stimulated groups. Additional results and analyses from OCT data (optic axis and DOPU) and the functional assessment tests (walking track, Von Frey, and nerve volume) are shown in the Supplementary Material.

### 3.3 Histological Findings and Correlation to PSOCT Results

**Fig. 7:**
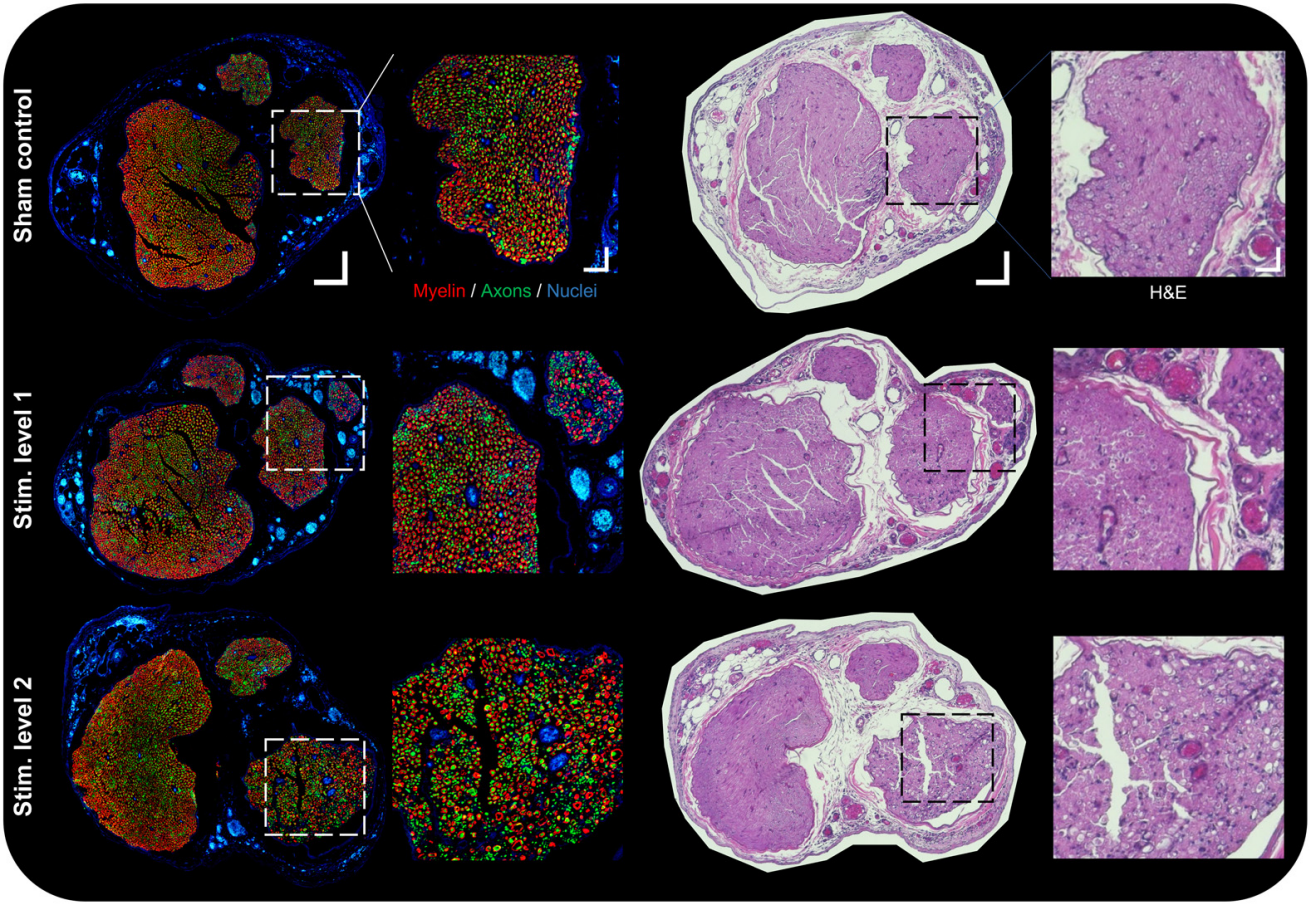
Histology co-registration and comparison. Representative IHC and H&E-stained sections from each stimulation group. Insets are expanded views of the fascicle nearest to the stimulation leads (right side). Sections were co-registered with PSOCT data and evaluated for signs of injury. IHC and H&E scale bars represent 125  μm (35  μm inset), acquired at 10×.

**Fig. 8:**
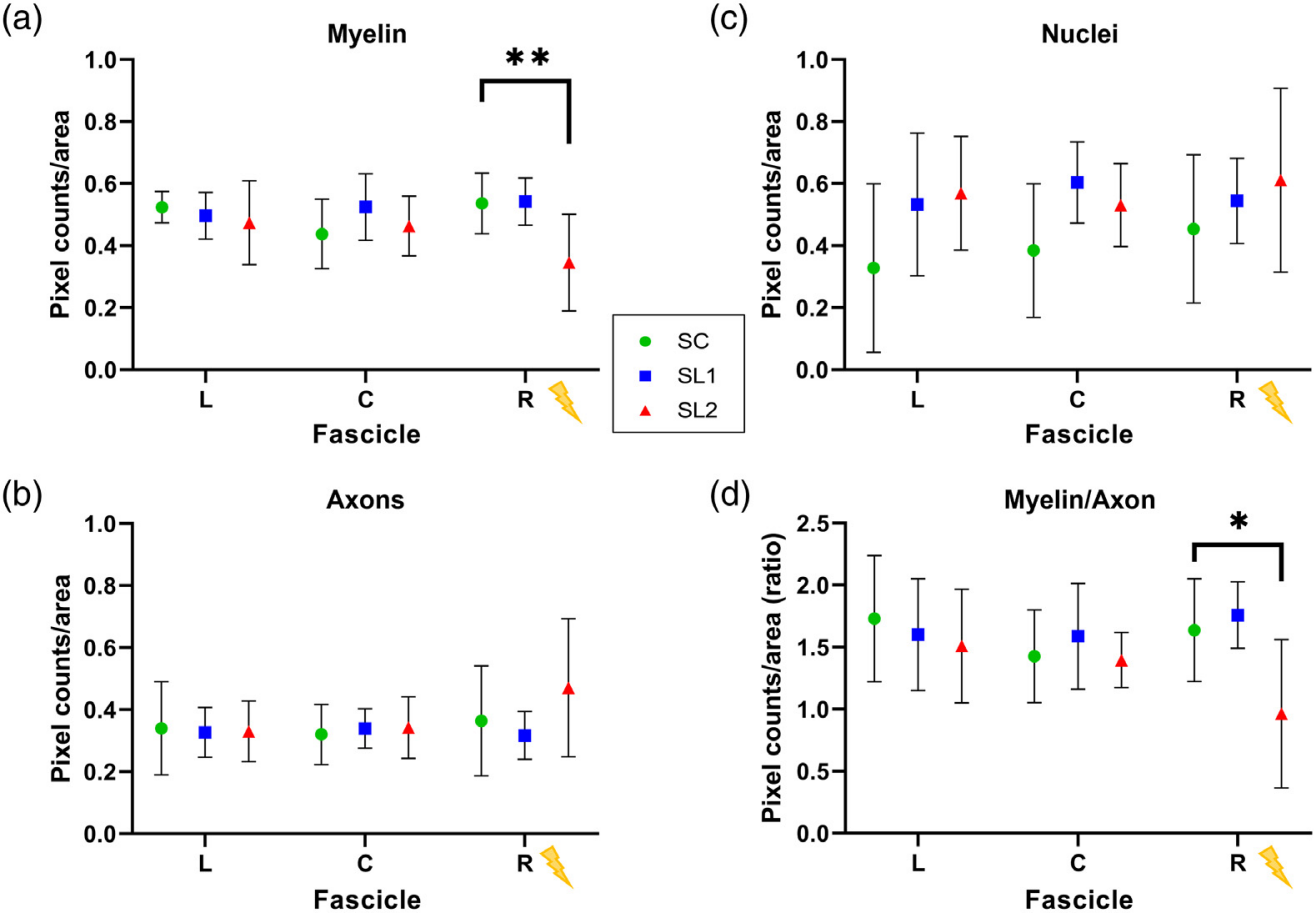
IHC image analysis results. Relative structural components are quantified in fluorescent IHC images by summing the fluorescent pixels within a fascicle ROI (D7 to D1) and comparing between stimulation groups. (a) Red pixels (myelin, SL2 **p<0.005), (b) green pixels (axons), (c) blue pixels (cell nuclei/DAPI), and (d) the ratio of myelin/axon pixel counts (SL2 *p<0.05). Left/center/right (L/C/R) denotes the fascicle position, with R closest to the deinsulated portion of the electrode. Error bars: ±SD. $\Delta \;\text{Metric}={\mathrm{avg}}_{\text{stim}\;\mathrm{grp}}(D7_{2D\;\text{fasc}}-D1_{2D\;\text{fasc}}$).

Representative IHC and H+E images from each group are shown in Fig. 7. In the IHC SC group, the fascicles appeared intact and healthy, with a regular distribution of fibers and blood vessels in each fascicle. In the stimulation groups, there is an increasing amount of disorder, manifested in irregularly shaped myelin sheaths, a reduction in myelination counts (red channel) and an uptick in axon pixel counts (green channel), especially closest to the stimulation lead in the rightmost fascicle, which is magnified and inset in the dotted boxed areas. IHC findings were quantified and plotted in Fig. 8, taking the same averaged D7 to D1 comparison as in Fig. 6, using two-way ANOVA and Tukey’s test. H+E images follow a similar trend to IHC data. The SC group showed intact and regular epineurium and perineurium boundaries. The stimulation groups seemed to have thicker regions in those boundaries due to the growth of additional and increased diameter blood vessels. In the stimulation groups, the increase in blood vessel counts and size was evident over the SC. Finally, the fascicle nearest the stimulation lead, especially in SL2, showed an increasing number of vacuoles with the stimulation group, which is a known marker of nerve injury.^53^

Overall, changes observed in OCT and IHC data followed the stimulation intensity. When comparing values from the stimulation group against the SC, there was (SL1/SL2): +4%/−309% (p<0.001, SL2) average change in phase retardation, and −79%/−148% reduction in optical attenuation correlated to a +1%/−36% (p<0.005, SL2) change in myelin pixels, −13%/+29% change in axon pixels, +20%/+35% increase in cell nuclei pixels, and +7%/−41% (p<0.05, SL2) change in the myelin/axon pixel ratio in the stimulated region of the rightmost fascicle.

## 4 Discussion

Building on our previous work to establish OCT angiography-based biomarkers of vascular and flow changes related to overstimulation,^34^ we sought to explore polarization-sensitive OCT imaging to detect and quantify changes to the birefringent properties of peripheral nerves during overstimulation. Unlike our previous OCT-A results, changes to the polarization properties of the nerve do not occur during stimulation. Rather, these biological effects follow the nerve injury and repair process. This study represents the first attempt to demonstrate PSOCT changes with overstimulation in the PNS.

The injury-repair process in the sciatic nerve was induced via electrical stimulation. The Shannon criteria states that k values above the threshold (k>1.85) are known to cause damage, at least in the CNS, with larger values imparting more severe damage.^12^^,^^41^ The k values used in this study were above that threshold and thus expected to cause damage (SL1 = 2.57 and SL2 = 3.17), and to simulate a scenario where stimulation does not cause obvious structural damage yet imparts functional damage to the nerve. In cases of device failure or serious electrical malfunction where more severe levels of stimulation are applied, significant reorganization of tissue and correspondingly larger changes in PSOCT metrics would be expected.

It is known that damaged peripheral nerves will undergo Wallerian degeneration,^11^^,^^54^ during which damaged axons and then myelin will degrade with help from macrophages and Schwann cells. This is an initial and necessary part of the peripheral nerve repair and regeneration process. With our PS-OCT methodology, it is possible to observe this process as it progresses. At D7, initial degenerative changes in myelin are expected and quantified with the OCT metrics—and compared to changes observed in IHC and H+E. Among the set of PSOCT metrics explored in this study, the tissue phase retardance appears to track with nerve myelination at baseline and demyelination (reduction in phase retardation) post-injury. Consistent with the nerve degeneration process, IHC data demonstrate a similar reduction in myelin pixel count in the fascicle closest to the stimulation electrode [Fig. 8(a)], as well as a slight increase (nonsignificant for our animal numbers) in cell nuclei pixels in all fascicles [Fig. 8(c)]. The pixels counting axons showed little change across fascicles or SLs [Fig. 8(b)], with a very slight nonsignificant increase in the SL2-R fascicle. These results follow expected changes at 7-days post-stimulation injury and postsurgical recovery, with myelin degradation. This result warrants further exploration of OCT metrics in relation to swelling and axonal splitting/sprouting within the stimulation region,^55^ especially in relation to the level of damage delivered to the tissue and when observed after a specific recovery period. It will be crucial to continue to link changes observed in OCT metrics to specific Shannon k values or other forms of nerve injury observed after “N”-days of recovery across a range of circumstances to observe different stages of degeneration, nerve repair or reorganization, and regeneration. This was performed previously in nerve crush models with a similar system,^35^ which tracked nerve recovery over 3+ weeks. An electrical stimulation-based model, as used in this paper, would likely follow a similar trajectory, though the type and extent of damage is quite different.

The imaging and stimulation platform (diagram in Fig. 1) was carefully designed and calibrated to ensure the measured imaging metrics correspond to a true biological change, as detailed in Figs. S2 and S3 in the Supplementary Material. Data analyses were similarly focused on changes within the fascicles and ignored changes within the epineurium or perineurium. The increasing SLs track closely with the observed damage (demyelination/IHC and increased vacuole count/H+E) and are most prominent in the rightmost fascicle in SL2. Von Frey and walking track analysis (Fig. S4 in the Supplementary Material) showed only minor functional effects, confirming that the extent of electrical damage was rather minimal overall and not clearly detected by these functional tests. Quantifying changes in hindlimb grip strength may be an interesting functional indicator of mild PNS injury in a future study. The animals were generally a bit more guarded with the operated leg, consistent with recordings in other studies.^56^ Similarly, nerve volume changes (Fig. S5 in the Supplementary Material) as calculated with OCT data did not show clear differences between experimental groups, likely due to the overlap of competing effects from surgical recovery and electrical overstimulation damage.^57^^,^^58^ By comparison, stimulation-induced changes to the optic axis were not observed (Fig. S8 in the Supplementary Material). This was expected, as the optic axis describes the orientation and alignment of nerve fibers, and this is unlikely to significantly change in response to the SLs applied in this study. The effects on changes in DOPU (Fig. S8 in the Supplementary Material) follow what is observed with phase retardance, with greater change in the stimulated nerves.

Existing methods to noninvasively assess nerve damage are imperfect. Fluorescence imaging is the most extensively used optical imaging technique to observe the structural and functional organization of nerves. However, fluorescence often requires an exogenous contrast agent, or one endogenously expressed through genetic manipulation, and an appropriate detection system. Some progress has been made to scale this technique to identify nerves during surgery,^59^ relying on an exogenous contrast agent and viewing system to visualize nerve location. Intrinsic optical imaging is another technique that can identify nerve activity, though similarly relies on voltage-sensitive dyes.^60^ Nerve conduction velocity tests are a standard clinical practice to check for nerve function^61^ but provide limited information of the location of finer nerves or the cause and extent of nerve damage. Research exploring the use of other standard medical imaging techniques, such as MRI and Ultrasound, is ongoing to identify lesions and impingement or blood flow issues.^62^ However, the resolution required to observe finer peripheral nerves in humans and more pertinently in animal models is not consistent in all MRI/Ultrasound equipment. Furthermore, many techniques cannot distinguish nerve tracts from surrounding tissue and instead rely on known structural cues. Interestingly, progress is being made for peripheral nerve MRI contrast agents.^63^ Ultimately, endpoint histology is the only reliable method to detect overstimulation or nerve damage, which is a completely invasive and destructive method to the site and nerve tissue.

OCT-based assessment of tissue offers many advantages. Tissue can remain intact and can be assessed immediately and without any stains or exogenous agents. OCT can also distinguish nerves from adjacent tissue, as the intrinsic birefringent structure of the nerve is distinct from that of muscle, fat, blood vessels, and other structures. If a nerve is accessible superficially, such as intravascularly, endoscopically, or near a major orifice or airway, noninvasive imaging is possible with modification to the OCT scanning arm. Otherwise, OCT has limited penetration depth (<3  mm), which requires the tissue of interest to be surgically exposed as was done in this study. Transcutaneous imaging of nerves is unlikely given this limitation. OCT is, therefore, well suited for integration into planned surgical procedures including neuromodulation device implantation. The optical metrics demonstrated here attempt to capture the range of expected biological changes in tissue from experimental stimulus. In this study, angiography and phase retardation measurements emerged to be most sensitive to vascular changes and demyelination as expected with this model of PNS injury and confirmed through experimental results. More broadly, these metrics reflect changes in optical signatures of tissue composition and ultrastructure. Specifically, the phase retardance, optic axis, and DOPU are independent metrics that each informs some biological change occurring as a result of experimentation. This response is dependent upon the specific disease or experimental model and related repair or regeneration mechanism in tissue, which varies across organ systems.

There are several limitations in this study. Angiography metrics calculate response over the entire nerve, as it is not straightforward to break up results by fascicle as with PSOCT metrics. However, an improved metric may take into account proximity to the electrodes and more closely examine the relationship between frequency and power (current) delivered to peripheral nerves. Results were collected at a 1-week post-recovery timepoint. Additional, later timepoints and greater stimulation levels would have been informative, perhaps at 2 weeks and with another much larger k-value serving as a positive control. However, the presented results demonstrate the capability to observe effects of these suprathreshold stimulation values at D7. For histological analysis, IHC and H+E were used. An additional contrast using osmium tetroxide staining would help to highlight the myelin sheaths as additional confirmation of the visualized changes.^64^ Collagen would similarly be a future target to explore,^65^ as PSOCT is sensitive to collagen remodeling, which occurs in tandem with myelin sheath degradation.^66^^,^^67^ Higher attenuation than expected in polarization-sensitive channels (phase retardation and optic axis) was observed overall in the study data. A PS signal is consistent over a short depth and will succumb to multiple scattering effects in any tissue, though the rate of degradation was greater than previously observed from a similar system.^35^ Although this does not impact the accuracy or validity or the results shown, only 70% to 80% of the total nerve visible on the reflectance channel can be quantified using PS-focused channels in the system currently. Investigation into the system hardware and experimental protocol to optimize light penetration and PS signal extraction are ongoing.

With a library of data over a range of stimulation values with corresponding histology and PSOCT metrics, this system could be used for a longitudinal assessment of nerve health, with histology performed terminally to confirm. With further testing and development, the impact would be that a substantially smaller number of animals would be necessary to observe equivalent processes. Apart from the application detailed in this paper, the platform can assist in surgical guidance, for example in mapping nerve tracts, especially when isolating finer branches that can be easily missed during delicate dissections or when exploring innervation points on organ systems. It can be likewise used to ensure that the blood flow is uninterrupted by either surgical technique or implanted device.

## 5 Conclusion

This work defines quantitative optical imaging-based metrics and methods for peripheral nerve stimulation and damage. The developed platform provides a test-bed for imaging and tracing fascicles in peripheral nerve bundles as well as their target ganglia, and understanding the biological mechanisms and effects related to electrical stimulation, ranging from testing different stimulation parameters to directly observing the downstream results and off-target effects of stimulation. The output of this system provides objective, quantitative measures of stimulation effect that can be used to assess safety, performance, and effectiveness of future neuromodulation medical devices.

## Code, Data, and Materials Availability

All data in this manuscript, including OCT and histological images, are available for download through Pennsieve (https://app.pennsieve.io/). OCT processing code is available online.^40^
