# Supplementary material for: Development of polarization-sensitive optical coherence tomography imaging platform and metrics to quantify electrostimulation-induced peripheral nerve injury in vivo in a small animal model
Source: Neurophotonics. 2023 Apr 17;10(2):025004. doi: 10.1117/1.NPh.10.2.025004 (PMC10109528; doi:10.1117/1.NPh.10.2.025004)
Supplement: Supplementary file 1 [file NPh_010_025004_SD001.pdf]

Development of polarization-sensitive OCT imaging platform and metrics to quantify electrostimulation-induced peripheral nerve injury *in vivo* in a small animal model (supplementary materials)

Guillermo L. Monroy[1], Mohsen Erfanzadeh[2,3], Michael Tao[1], Damon T. DePaoli[2,3], Ilyas Saytashev[1], Stephanie A. Nam[2,3], Harmain Rafi[1], Kasey C. Kwong[2,3], Katherine Shea[4], Benjamin J. Vakoc[2,3,5], Srikanth Vasudevan[1*], Daniel X. Hammer[1*]

[1] Division of Biomedical Physics, Office of Science and Engineering Laboratories, Center for Drugs and Radiological Health, U. S. Food and Drug Administration, White Oak, MD 20993 USA
[2] Wellman Center for Photomedicine, Harvard Medical School and Massachusetts General Hospital, Boston, MA 02114 USA
[3] Harvard Medical School, Boston, MA 02115 USA
[4] Division of Applied Regulatory Science, Office of Clinical Pharmacology, Office of Translational Science, Center for Drug Evaluation and Research, U. S. Food and Drug Administration, White Oak, MD 20993
[5] Division of Health Science and Technology (HST), Massachusetts Institute of Technology, Cambridge, MA 02139 USA

*Correspondence E-mail: srikanthvasudevan1@gmail.com, Daniel.Hammer@fda.hhs.gov

1. Surgical protocol

For a magnified view of the sterile field, a surgical microscope was used with a sterile pad and warming mat to stabilize animal body temperature during procedures. Before each use, titanium surgical tools were sterilized in an autoclave, and temperature sensitive components, such as the nerve stabilizer, were sterilized with ethylene-oxide (C_2H_4O) to avoid degradation from repeated thermal cycling. Other surgical supplies and reagents (mats, gloves, saline, etc.) came pre-sterilized from the manufacturer. To prepare for surgery, animals were temporarily anesthetized with 3% isoflurane in O_2 in an induction chamber. An intraperitoneal injection of ketamine (75 mg kg^{-1}) and dexmedetomidine (0.25 mg kg^{-1}) was administered, which kept animals fully anesthetized during the entire imaging session. During either day of imaging, if any factors indicated insufficient anesthesia, a booster dose was given (typically ¼ of the original dose) to extend anesthesia to complete imaging and proper surgical closure or euthanasia.

1.1 *D1 surgical protocol*

After injection, the experimental (left) leg was shaved and disinfected using alcohol and betadine surgical scrub (povidone-iodine 7.5%, Purdue Products, USA). Puralube was applied to the eyes to prevent dryness, and subcutaneous injections of saline (3 mL, hydration), and Meloxicam (2 mg kg^{-1}, analgesic) were administered. Adequate sedation was confirmed with a toe and tail pinch test. Sciatic nerve exposure surgery then followed in a sterile surgical field following aseptic technique. Generally, an incision was made into the skin along the thigh to expose the biceps femoris. The muscle was incised and spread to reveal the sciatic nerve, which was mobilized from the surrounding fascia. If needed, a propylene 4-0 suture (Oasis Inc., USA) was inserted to prevent any surrounding tissue (primarily muscle and skin) from obscuring the sciatic nerve during imaging.

After imaging, the incision was closed. Any sutures to keep the nerve exposed were removed and tissue rehydrated with saline. The Biceps Femoris was sutured using single interrupted stitches spaced approximately 10 mm apart along the length of incision. The skin was stapled closed and antibiotic ointment was applied to the incision. Gentamicin (8 mg kg^{-1}), Meloxicam (1 mg kg^{-1}), and saline (3 mL) were given subcutaneously. Finally, the animal was given an intraperitoneal injection of Atipamezole (0.5 mg kg^{-1}) and brought out of anesthesia. Post-operative recovery consisted of close monitoring for 1-2 hours with a gentle heating pad and saline as needed. Post-surgical recovery was closely monitored, with inspection in animal housing twice a day for 5 days. Meloxicam (1 mg kg^{-1}) was provided for the first 2 consecutive days post-surgery, as well as nutrient gel and gentle heating pads for comfort as needed.

1.2 *D7 surgical protocol*

On D7, after anesthesia (as in D1), stitches on the previously operated (left) leg were removed sequentially and the skin and muscle were reopened using blunt dissection for imaging. Next, the procedure outlined on D1 was repeated on the previously untouched contralateral (right) leg. After follow-up imaging was completed on both legs, the sciatic nerve was harvested from both treatment and contralateral legs, and animals were euthanized with an intracardiac injection of pentobarbital (200 mg kg $^{-1}$). Each nerve was separated into 3 segments, relative to where stimulation was applied, and placed into separate vials with different fixative agents for histology. Distal and proximal segments were ~2-3 mm portions, downstream and upstream from the stimulated nerve region (i.e., closer to hip or limb extremity) and known to be outside the OCT scan area. The median segment was ~7-10 mm and known to contain the stimulated nerve region and have confirmed overlap with the PSOCT image data.

2. Imaging platform and experimental setup

The stimulation and imaging platform shown in **Fig. S1** is based on a 1310-nm fiber-based swept-source PSOCT system (Nine Point Medical, USA). The system employs a 36-mm effective focal length scan lens objective (LSM03, Thorlabs, USA) with a polygonal mirror 110-nm bandwidth swept-source laser source (HSL-200-50LC, Santec Corp., Japan), which provides an axial and lateral resolution of approximately 7 and 20 micrometers (in air). To measure tissue birefringence independent of tissue optical axis, this system modulates the input polarization state of light on alternating A-lines [36] using a custom-fabricated semiconductor-based polarization modulator (Boston Applied Technologies, USA). OCT-A scans are simultaneously collected with PSOCT data by taking 5 sequentially repeated scans at each physical location. The system runs at an A-scan rate of 50 kHz, with one full volumetric dataset recorded in about 1.8 minutes. Before each day of use, the system was recalibrated by equalizing signal at dual-balanced receivers to ensure consistent and optimal performance. Testing has shown it to be stable over a typical imaging session, which is detailed in a later section.

For stimulation, two Teflon-coated platinum/iridium hemi-cuff stimulation electrodes were installed into the nerve channel (Microprobes for Life Science, USA). Both electrodes had a diameter of ~0.05 mm, with de-insulated regions of 0.2 mm (positive) and 0.4 mm (negative) within the nerve stabilizer holder. Additional information regarding the nerve stabilizer, including material composition and design, can be found in a previously published paper [34]. A MATLAB-controllable precision AC/DC current source (6221, Keithley Instruments, USA) delivers customizable stimulation waveforms. Lastly, a sterile, thin piece of plastic film, approximately the dimensions of the nerve channel, was placed over the nerve to reduce back reflections and prevent dehydration effects over the imaging period.

The system has several application specific features that were developed or updated for this work. A green laser collinear with the PSOCT scan beam was added using a 99:1 coupler in the sample arm to mark the imaging field on the nerve. In tandem with a 3-axis motorized stage (MLJ150 Thorlabs, USA), the animal and nerve target can be quickly positioned for imaging. Underneath a sterile drape, a heating pad controlled by a thermometer (inserted rectally with petroleum jelly) helps to regulate subject core body temperature during experimentation. A nearby syringe pump administered sterile saline (0.05 mL/min) over the course of experiments to keep tissue hydrated. A custom-designed 3D-printed nerve stabilizer was modified from previous work [34] to include PSOCT calibration phantoms [37-39]. The stabilizer supports the nerve during imaging to reduce motion artifacts from breathing and electrical stimulation.

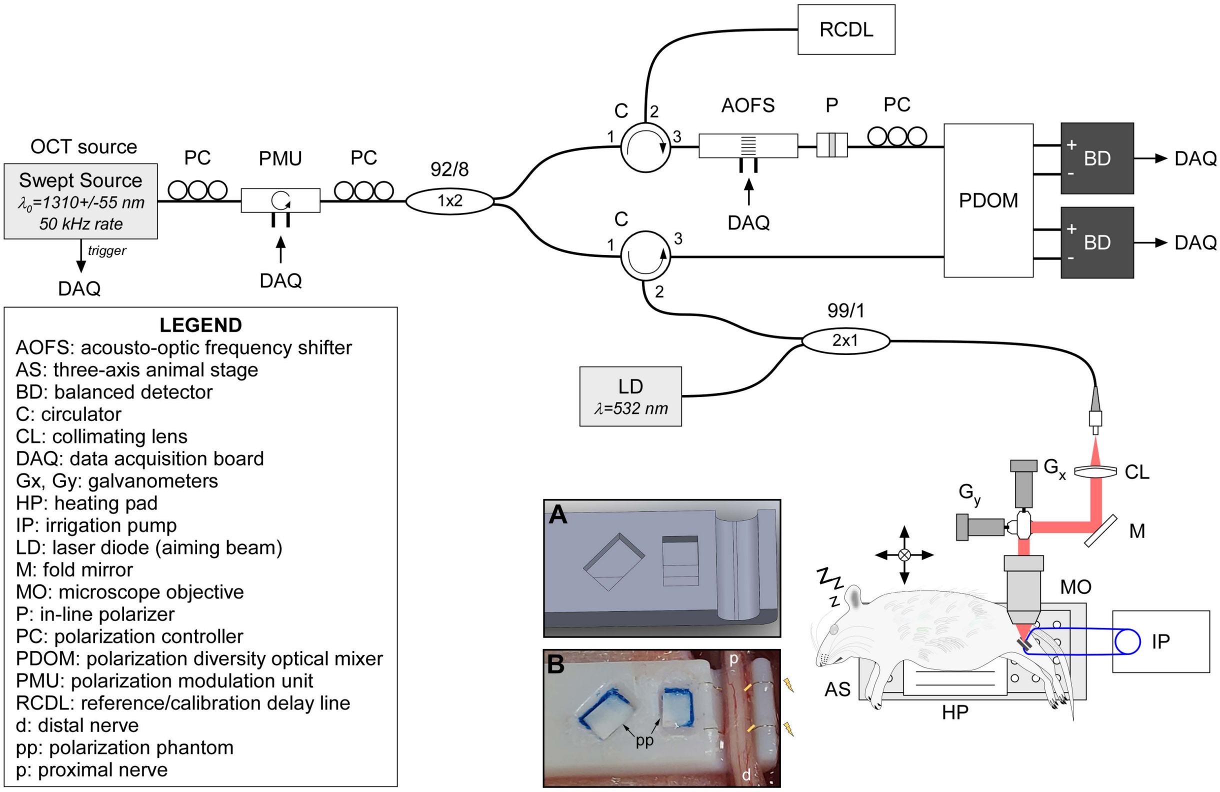

Fig. S1 (duplicated from Fig 1 in the main text). Optical coherence tomography (OCT) Imaging – full system layout. This custom-built swept-source polarization-sensitive OCT system was designed to capture volumetric scans of the sciatic nerve during electrical stimulation. This system noninvasively extracts tissue features (structure, birefringence, and perfusion) without stains or dyes. **A**: Nerve stabilizer 3D-model; **B: S**tabilizer with sciatic nerve *in situ*. De-insulated portions of the electrodes are noted with yellow bars, nearest to the rightmost fascicle.

To record data, the system uses a customized scan pattern that collects 592×1776×2560 pixels (2.5×6.5×2.0 mm / X×Y×Z (depth)) with 5 repeats to collect OCT-A scans and averaging for PSOCT. 296 out of 1776 pixels in the Y dimension are dedicated to imaging calibration phantoms before and after nerve imaging, which serves as an internal stability check during imaging and for later calibration. The remaining 1480 pixels cover the nerve channel. Initial alignment scans help to rapidly position the nerve and phantoms using the 3-axis stage. This alignment procedure was performed before each dataset was acquired.

For PSOCT image calibration, a pair of polarization phantoms are embedded in the nerve stabilizer and manually set at 0 and 45 degrees (X-Y rotation), offset from the imaging axis by 10 degrees (X-Z rotation) to reduce back-reflections during imaging. The phantoms are used as fixed external markers to calibrate the optic axis of the imaging system, much like a Color Checker is used to calibrate the color spectrum in film or digital media [68]. Once normalized in post-processing, PSOCT measurements are absolute and can be directly compared to one another, even when collected on different days or different imaging systems. Without the phantoms, or if only a single phantom were used, only a relative difference between two polarization values within the same image could be compared between datasets. **Fig. S2** details the testing and validation used to measure the birefringence of calibration phantoms prior to beginning this study. OCT scans of a three phantom array were taken across two days to ensure consistent and accurate measure of phase

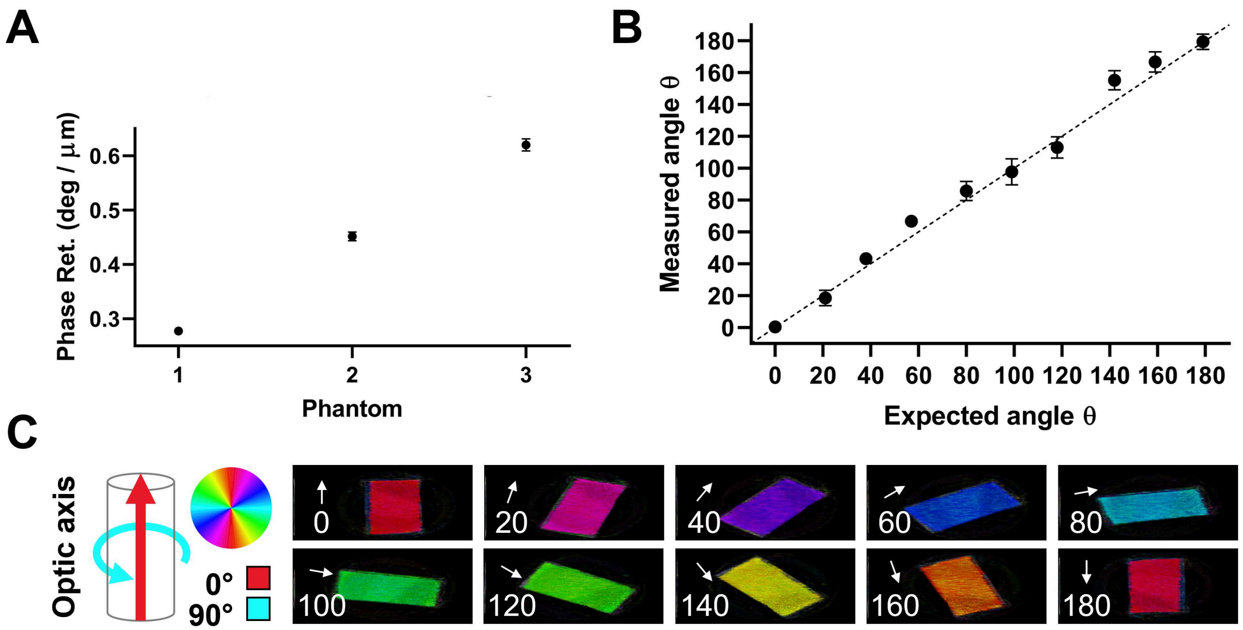

Fig. S2. OCT System measurement accuracy and repeatability. Polarization phantoms serve as an external calibration reference for birefringence and were used to assess the repeatability of system precision and accuracy. **A**: 3 phantoms were measured 6 times over the course of 2 days to test phase retardation measurement variability under different alignment and system conditions. **B**: Optic axis measurement precision and accuracy. A phantom was rotated in 20° intervals across 0°-180° to ensure accurate measurement of optic axis orientation. **C**: PSOCT images of a polarization phantom at various stages of rotation, demonstrating the color dependency with angle. Error Bars: SD.

retardation and optic axis values. **Fig. S2A** shows minimal variation (<**2%)** for each phantom over time, two orders of magnitude below measurement values (mean +/- std. dev. values plotted). Similarly, **Fig. S2B** shows optic axis performance was linear (R^2 = 0.9882 with average deviation of +/- 4°) over several acquisitions. These results demonstrate good system performance and define measurement sensitivity for reproducibly quantifying the polarization properties of birefringent tissue targets.

Bands of Fontana (BOF), accordion-like folds inherent to a compressed peripheral nerve [69], can obscure the appearance of the sciatic nerve when the leg is in a relaxed state. To reduce the appearance of BOF, a rodent leg extender (RLE) was created. The RLE, shown in **Fig. S3**, gently and safely applies force around the ankle using a rubber band to pull the leg and sciatic nerve into its naturally extended position. The appearance of the BOF are heavily reduced in PSOCT images, limiting their influence on the image analysis and quantification.

To manage and process PSOCT data, customized MATLAB processing scripts were developed and packaged into turn-key scripts that took raw data as input and automatically generated processed images. These scripts at first ran serially in batch mode, which included recalibration and processing of multiple structural/OCT-A/PSOCT data channel volumes [35, 70-71]. A major task in this project was to combine many disparate components into a unified process, which improved overall runtime by 50%. This program was later converted to utilize parallelized GPU-based processing [40], with a final runtime of approximately 1 hour per dataset. PSOCT processing parameters that control the polarization-related channel reconstruction were calibrated for

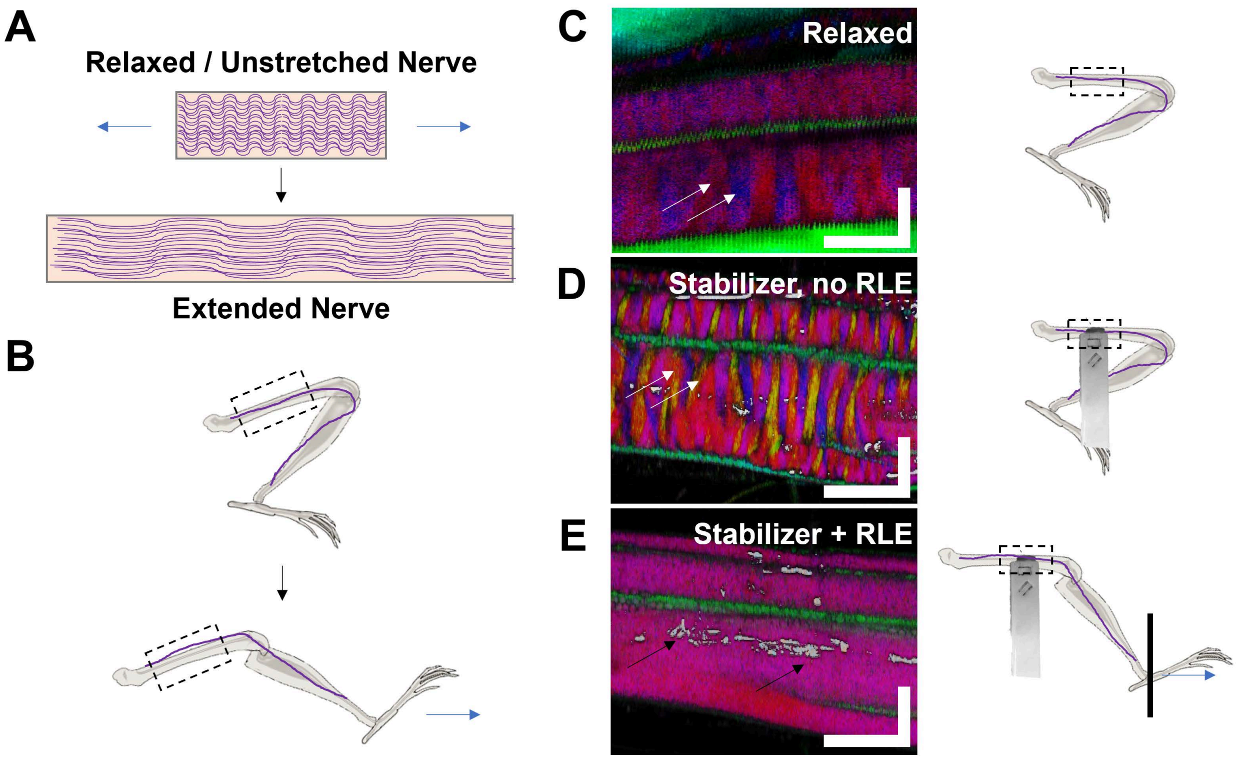

Fig. S3. Reducing Bands of Fontana in OCT images. The rodent leg extender (RLE) aims to reduce striations in OCT images, which are likely created by folded oblique bands in peripheral nerves called Bands of Fontana (BOF). **A**: The sciatic nerve depicted in unstretched / relaxed and extended conditions. **B**: To reduce the appearance of BOF, an RLE was developed to gently extend both the leg and sciatic nerve to its full natural extension during imaging. **C**: Rodent leg observed in a free and relaxed state, with corresponding *en face* BwOA projection. The sciatic nerve is shown primarily in purple/pink, with 'zebra striping' (white arrows) caused by BOF. **D**: The stabilizer mechanically supports and stabilizes the nerve during imaging. However, the BOF are still present (White arrows). **E**: With the nerve stabilizer and RLE in place, the appearance of BOF in OCT images are significantly reduced. Some strong back-reflections causing saturation in this panel (Black arrows) and are visible due to the lack of plastic wrap covering on the nerve. All scale bars (white, solid) represent approximately 500 µm x 500 µm. (Note: In surgical images, additional tissue removed for RLE and other testing purposes - study cohort left as intact as possible).

this system. Proper tuning of spectral binning and localized spatial filtering parameters improves the quality of local phase retardance calculations, reduces polarization mode dispersion, and ensures reconstructed data accurately represents tissue morphology observed in histological sections. These parameters are determined by polarization-dependent noise specific to this system, as well as the optical properties of tissue and its anatomy. Poorly tuned parameters may create artifacts that appear as false discontinuities or interfaces on phase retardation maps or result in a loss of dynamic range and fidelity. A finalized set of parameters was manually determined and tuned across several datasets in this study for verification. Then, every dataset was re-processed with the final optimized set of processing parameters before analysis.

3. Study results and analysis

Figure S4 presents behavioral results from this study. Walking track analysis of the SL1 and SL2 groups showed a non-significant but identifiable decrease in the mean TFI and SFI with stimulation group. The Von Frey data is inconclusive. Qualitatively, animals in the stimulation groups seemed to be guarded with their stimulated legs and seemed to overcompensate by putting more weight on the contralateral leg. This observed behavior is captured in the data for SL1. However, there seems to be little change in withdrawal force in SL2. The data shows the averaged D7-D1 per group and fascicle, with two-way ANOVA and Tukey's test. The branches of the sciatic nerve that were impacted that may or may not impact these tests. Loss of grip strength is one sign of peripheral nerve injury [72], and apart from SFI, quantifying changes in hindlimb grip strength may be an interesting functional indicator of mild injury in the future.

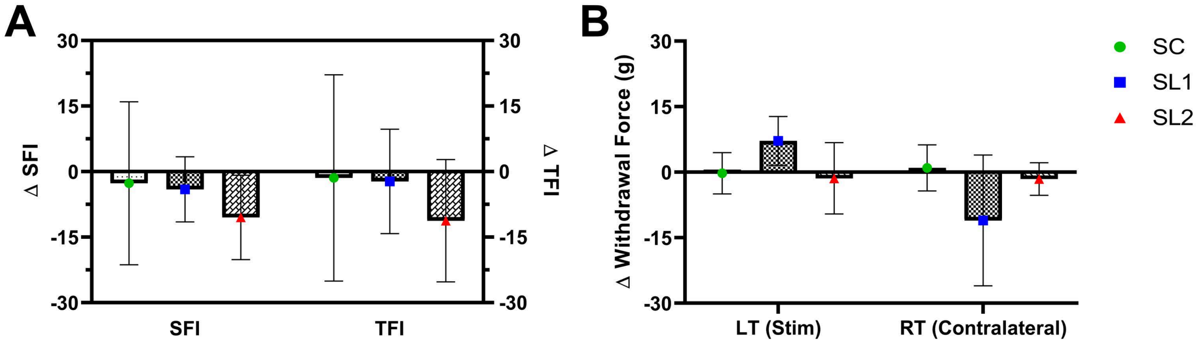

Fig. S4. Functional and behavioral test results. Data for each metric was calculated and averaged across stimulation groups $Avg_{Stim\ Grp}(D7_{2D\ Fasc} - D1_{2D\ Fasc})$. **A**: Walking Track results show that with increasing stimulation level, both SFI and TFI values decrease, though the change does not reach statistical significance. **B**: Von Frey results show that a decrease in the applied force is needed to induce a response in the SL1 group in the left/stim leg, with a corresponding decrease in the contralateral leg. Error Bars: SD.

Nerve segmentation volume analysis (**Fig. S5**) showed that on average, overall nerve volume as measured by PSOCT slightly increased in the stimulated cases. As discussed in the main text, these effects were not statistically significant.

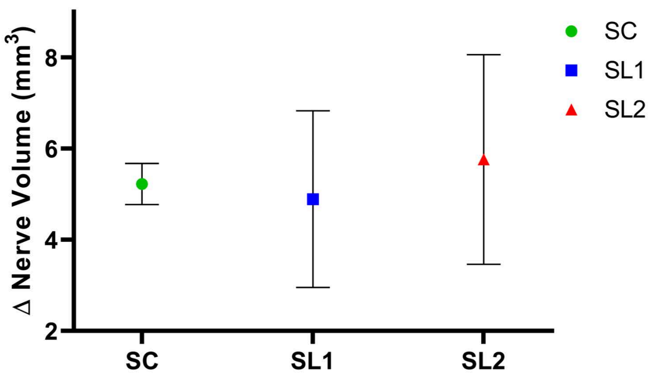

Fig. S5. Nerve volume segmentation. Results $Avg_{Stim\ Grp}(D7_{2D\ Fasc} - D1_{2D\ Fasc})$ indicate a non-significant increase in overall nerve volume with stimulation. Error Bars: SD.

Figure S6 (next page) shows representative imaging data from all groups. **Fig. S6A+B** show SC, **Fig. S6C+D** show SL1, and **Fig. S6E+F** SL2. Scanning artifacts in **Fig. S6A** distorted the baseline scan, though other timepoints do not have this issue. Changes in PSOCT data were quantified and described in the results and discussion sections.

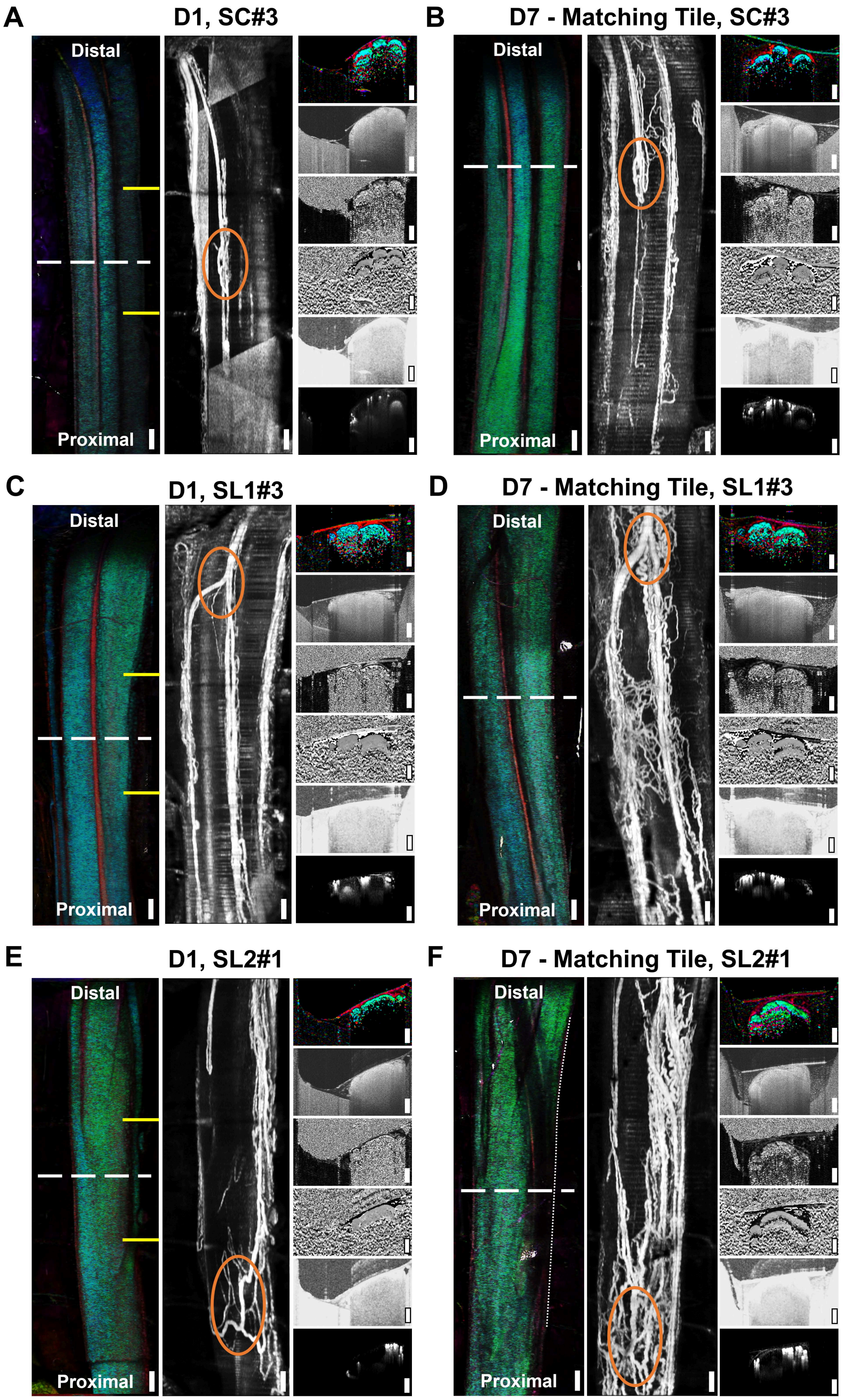

Fig. S6. Representative imaging results across stimulation groups. A: SC#3 Baseline, including *en face* projections of the birefringent-weighted optic axis (BwOA, Left), nerve perfusion map (Center), and cross-sectional scans (Right) from all data channels. Processing artifacts (Center) partially obscure the edges of this particular scan (geometric shadowing). **B:** Day 7, SC#3, showing changes after a 7-day recovery period with co-registered data from part B. **C:** Day 1 Baseline and **D:** Day 7, (SL1#3), **E:** Day 1 S2 scan, and **F:** Day 7, (SL2#1), with similar layout as in panels A and B respectively.

Note: Yellow lines mark stimulation region (Day 1). Orange circles demonstrate one perfusion map cue for co-registration. White-dotted lines denote cross-sectional scan location and visualize co-registration offset. Cross-sectional scans: (Row 1) structural, BwOA, and Angiography/Perfusion (corresponding to the *en face* projections) and (Row 2) phase retardation, optic axis, and degree of polarization. Distal and proximal labels indicate sciatic nerve orientation. Scale bars (White, solid) represent 500 μm in depth.

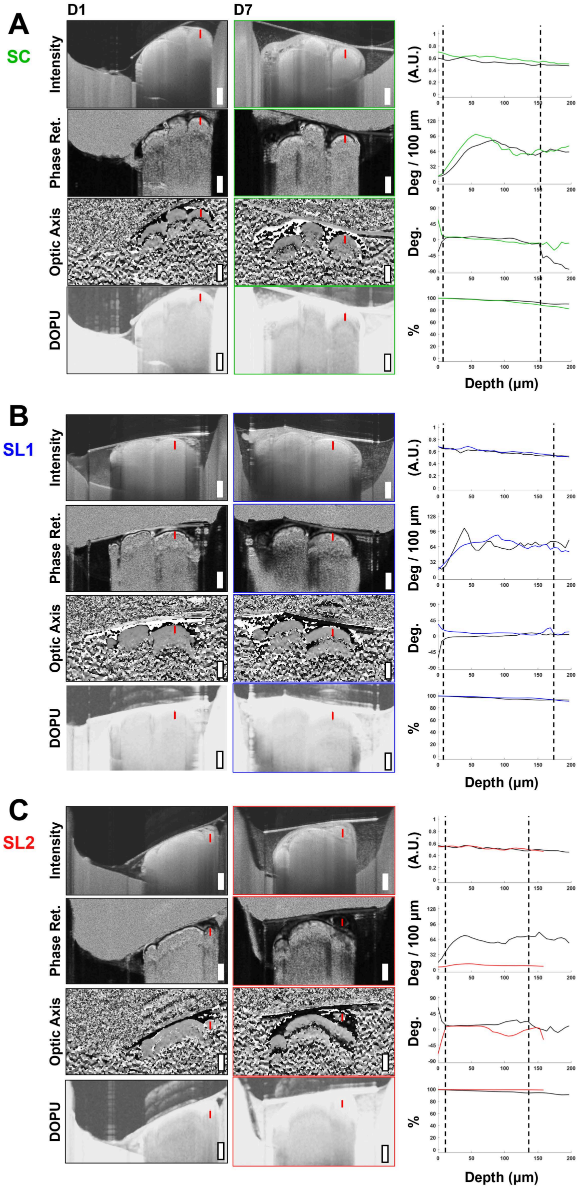

Fig S7: Representative PSOCT-2D and 1D depth profiles across stimulation groups. 2D cross-sectional images from the center resultant frame (25-frame average) from the datasets in Fig S6 displayed with 1D depth profiles from the center region of the rightmost fascicle, nearest the stimulation electrode. 1D profiles are provided to demonstrate depth-resolved analysis of nerve structure. Differences observed between stimulation groups, shown in main text Figs 6 and 8, can be visualized here.

Note: Red lines in 2D cross-section denote location of 1D depth plot. Scale bars (White, solid) represent 500 µm in depth. Dotted black lines denote the valid sampling depth determined by fascicle masks in PS-sensitive channels, as shown in Fig 4B, to prevent analyzing noise due to the observed reduction in PSOCT metrics over depth. Complete 2D fascicle area is used for analysis for Fig 6.

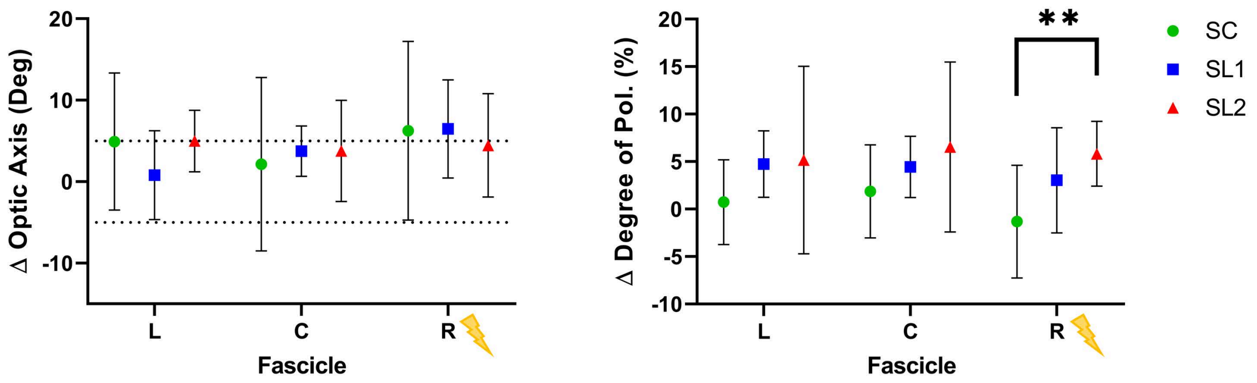

Fig. S8. Additional OCT metric results – Optic axis and degree of polarization change after 1 week. $Avg_{Stim\ Grp}(D7_{2D\ Fasc} - D1_{2D\ Fasc})$. **Left:** Measured differences in the optic axis channel are ambiguous at these stimulation levels, as they fall within the measurement sensitivity of the system (+/- 5 degrees). **Right:** Degree of polarization values increase with stimulation levels across the nerve, with the greatest change between groups seen in the rightmost fascicle (SL2 ** = $\mathbf{p < 0.01}$). Error Bars: SD.

Figure S8 shows additional results from PSOCT data, optic axis and degree of polarization, with averaged D7-D1 values by group and fascicle, and two-way ANOVA and Tukey's test for significance. For optic axis, most measurements are within or near +/- 5 degrees, which is the approximate measurement sensitivity of the system. Due to the weak stimulation levels used, overall tissue alignment did not change, even in the stimulated groups, nor was expected as the bulk nerve fiber orientation remained largely intact. For comparison, nerve crush injury causes substantial changes in these signals at the injury site [35] and is consistent with the severity of nerve injury. DOP is another metric that relates the continuity of the polarization properties in the tissue ROI. Results in DOP follow the general trend observed in other metrics, with an increase in the DOP value with stimulation group. SL1 shows a slight but non-significant increase in mean value in all fascicles over SC, and SL2 shows a similar slight increase over SL1, with significance in the rightmost fascicle.

While this manuscript focuses on specific details of neurostimulation, it can be taken as a complete demonstration of the utility of the PSOCT technology. The system was designed to be modified and deployed for related imaging applications in neuromodulation, such as mapping the central and peripheral nervous system and observing effects of stimulation on organ systems. We are interested in translating the imaging platform and disseminating the technology to the greater neuroscience community. See related research at https://commonfund.nih.gov/SPARC.
